# Transcriptome-based signature predicts the effect of taxol in serous ovarian cancer

**DOI:** 10.1371/journal.pone.0192812

**Published:** 2018-03-01

**Authors:** Shunyu Hou, Jianrong Dai

**Affiliations:** The Affiliated Suzhou Hospital of Nanjing Medical University, Suzhou, Jiangsu, P.R. China; University of South Alabama Mitchell Cancer Institute, UNITED STATES

## Abstract

Taxol is a widely used chemotherapy drug used clinically for ovarian cancer, although the response to Taxol among individuals varies due to the heterogeneity among ovarian cancer patients. In this work, we analyzed differences in the prognostic effect of gene expression and Taxol usage in the Cancer Genome Atlas (TCGA) dataset and identified specific genes associated with the Taxol effect. Using the Cox regression model, a risk model (Taxol score) was developed to assess the outcome of ovarian cancer patients who underwent chemotherapy with Taxol. According to the results, survival was significantly associated with the Taxol score. Moreover, the patients in the high and low Taxol score group had different responses to Taxol. This result was further validated in another two independent datasets. The correlation between clinicopathological indicators was also analyzed, and we determined that the Taxol score is not associated with age, pathological stage, or Taxol treatment, while there was significant correlation with tumor size and grade. Gene Set Enrichment Analysis (GSEA) showed that various signaling pathways including ECM receptor, drug metabolism and ascorbate metabolism pathways were significantly enriched in the high Taxol score group. Collectively, these results indicate that the model is robust for predicting the effectiveness of Taxol by reflecting the various cell statuses of serous ovarian carcinoma.

## Introduction

Ovarian carcinoma is one of the most common causes of cancer related death[1]. It was estimated that there were 52,100 new cases and 22,500 deaths in 2015 according to a recent cancer statistic in China [2]. Among the subtypes of ovarian cancer, serous ovarian carcinoma comprises the majority of the cases. Since radiation is not suitable for serous ovarian carcinoma therapy and targeted drugs have not been widely used, chemotherapy is currently the major treatment method for serous ovarian carcinoma after surgery. Among the drugs used for chemotherapy, Taxol is one of the most important drugs for therapy.

Due to the heterogeneity of ovarian serous carcinoma, Taxol resistance was detected in a large proportion of patients. This allowed for biomarkers and genes associated with Taxol resistance to be identified. For example, high expression of forkhead box protein M1 (FOXM1) was shown to contribute to paclitaxel resistance and correlated with survival of ovarian carcinoma[3]. Inhibition of the Il6-STAT3 axis also had an effect on Taxol resistance in ovarian cancer cells[4]. The clinical significance of Cluster of Differentiation 44 (CD44) up-regulation was evaluated, and it is was also shown to be associated with Taxol resistance and the prognosis of ovarian cancer[5]. In addition to protein-coding genes, miRNAs were also widely identified and reported to contribute to Taxol resistance[6]. MiR-197 was reported to predict survival of ovarian cancer patients via the regulation of Taxol resistant ovarian cancer cells. Additionally, exosomal transfer of stroma-derived miR21 was shown to confer paclitaxel resistance in ovarian cancer cells through targeting of APAF1.

Though efforts have been devoted to discovering novel biomarkers for guiding chemotherapy using Taxol, none of these biomarkers were used clinically due to their low performance. Taxol resistance is related to multiple signaling pathways; therefore, a single biomarker is not sufficient to predict the effectiveness of Taxol. Transcriptome-based models utilize the expression level of multiple genes in various pathways and develop a model to guide prognosis and therapy[7–10]. Since this modeling incorporates a variety of cell statuses, including cell adhesion and immune cell infiltration, it has been emphasized and has become prevalent in many cancer types.

In this article, genes related to survival and Taxol resistance were identified from the TCGA dataset. Using the Cox multivariate regression model, a scoring system was developed to evaluate the effectiveness of the model. The Taxol score was significantly associated with survival; patients with a low Taxol score had showed better survival when they underwent Taxol chemotherapy, while those with a high Taxol score did not show better survival when they underwent Taxol chemotherapy. These observations were confirmed in other validation datasets. The correlation between the Taxol score and clinicopathological indicators was analyzed, and KEGG pathways associated with Taxol score were identified.

## Materials and methods

### Sample enrollment and pre-processing of data

The samples in TCGA, GSE63885[11] and GSE9891[12] with records of Taxol treatment were enrolled in this study. Recurrent and metastatic samples were excluded from this study. The expression matrix of the TCGA dataset was downloaded from UCSC Xena (xena.ucsc.edu), and the expression was transformed to log 2 RFPM values using the original data. The Z-score was calculated for each gene across the samples. The genes expressed in less than 50% of the samples were excluded. The raw expression data of GSE63885 and GSE9891 were downloaded from GEO (www.ncbi.nih.gov/geo). After background correction and normalization, the expression values were z-score transformed to remove the platform bias. The information of cohorts was listed in S1 Table.

### Gene selection and model development

To exclude genes that contribute to the survival and were independent from Taxol response, correlation analyses between gene expression and survival of ovarian serous carcinoma patients were implemented in the TCGA-Taxol receiving group and the TCGA-Taxol depleted group. Genes significantly associated with survival in the TCGA-Taxol receiving group (p<0.05) while not significantly associated with survival in the TCGA-Taxol depleted group (p>0.05) were selected as candidate genes. All the combinations of the candidate genes were identified, that means all combinations from ${⋃}_{1}^{n}C_{n}^{i}$ was tested (where n refers to the candidate genes and C indicates the combination, and i is from 1-n), and we developed a risk model using Cox multivariate regression with each combination using the following formula and each combination,

$$\mathrm{Taxol}\;\mathrm{score}=\sum_{i}^{n}c_{i}x_{i}$$

where c_i_ is the coefficient resolved and x_i_ is the relative gene expression level. The survival differences between those with a low-score and those with a high-score were compared using Kaplan-Meier survival analysis. The combination model with the smallest p value was selected as the optimized combination.

### Statistical analysis

Survival difference analysis, Cox multivariate regression, and univariate regression was carried out using the “survival” package on the R platform (www.r-project.org). The heatmap of candidate gene expression was plotted using the “pheatmap” package. Gene Set Enrichment Analysis was implemented using java software[13], with TCGA gene expression matrix and high-score/low-score separation using the median Taxol score as a cutoff.

## Results

### Candidate gene selection and model development

Taxol-resistant genes were selected using the following criteria in the TCGA dataset. First, the expression levels of candidate genes that were significantly associated with survival in the Taxol-receiving subgroup (patients who received Taxol therapy) according to the Cox univariate regression (p<0.05) were determined. Second, the expression levels of candidate genes that were not significantly associated with survival (p>0.05) in the Taxol-depleted group (patients who did not received Taxol therapy) were determined using Cox univariate regression to exclude to probability that the gene is a prognostic gene for ovarian cancer but not involved in Taxol resistance. Thirteen genes were then selected as candidate genes for model development. To eliminate redundancy and optimize the panel, all combinations of these thirteen genes were enumerated. The Cox multivariate model was developed using these combinations to calculate the Taxol score in the Taxol-receiving subgroup. Using the median Taxol score value as a cutoff, the survival difference between those with a high Taxol score and a low Taxol score was compared, and the p values were calculated using Kaplan-Meier survival analysis. Finally, a combination of six genes (CLIP2, LAX1, PCK2, THOC1, LEPR, GSTZ1) was used for model development, and the Taxol score was calculated as follows:

Taxolscore=(0.114148691*CLIP2)+(‑0.190271239*LAX1)+(‑0.157333524*PCK2)+(‑0.151729207*THOC1)+(0.146653652*LEPR)+(‑0.092779415*GSTZ1)

where the gene symbol represents the relative expression level. Genes with positive values suggest that these genes contribute to the Taxol resistance, and vice versa.

### Model performance in the TCGA dataset and validation dataset

**Fig 1:**
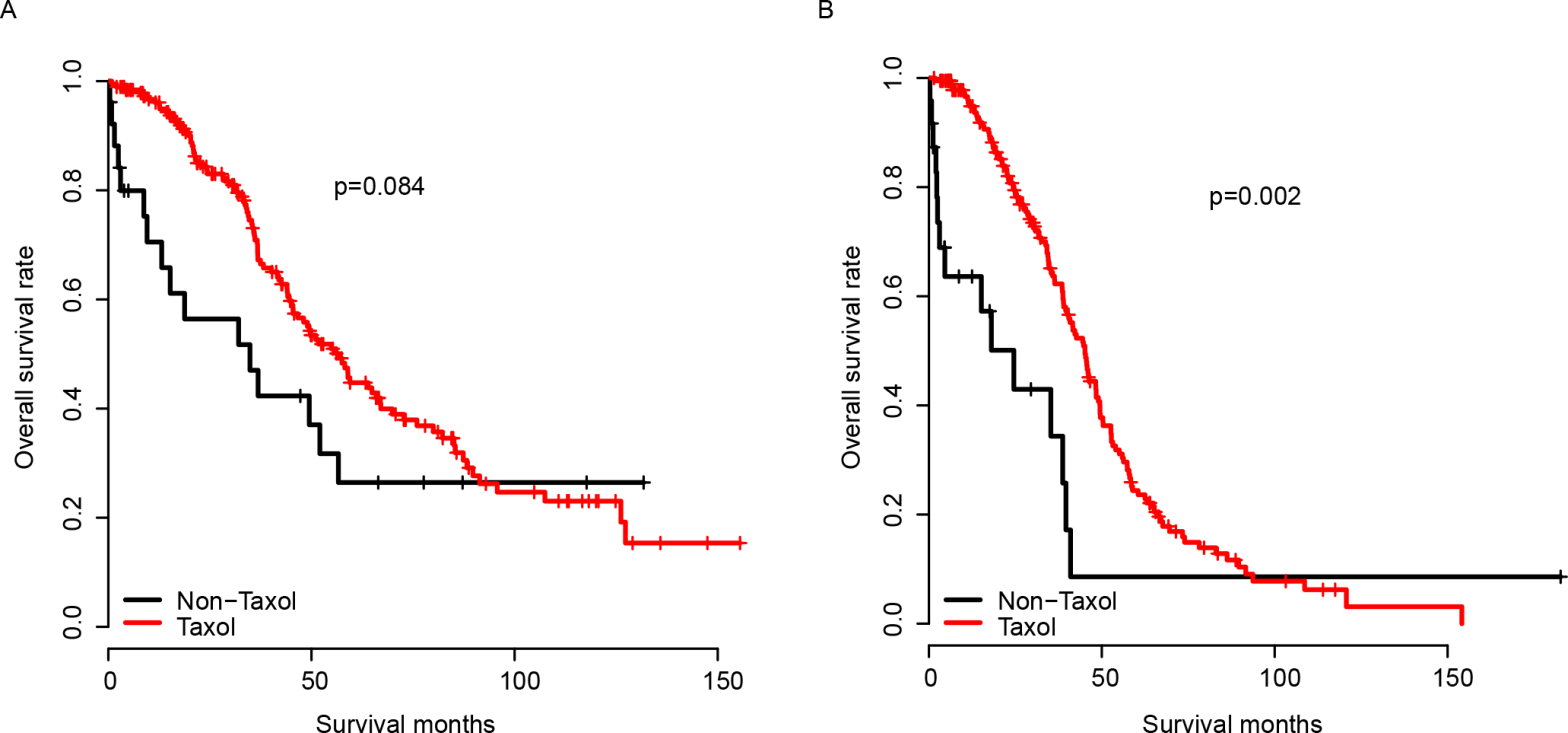
The performance of Taxol score in the training dataset. The survival of Taxol-receiving and Taxol-depleted groups was comparable in the high Taxol score group (A), while survival of the Taxol-receiving group was significantly better than the Taxol-depleted group in the low Taxol score group (B). The Taxol and Non-Taxol group refer to patients received and did not received Taxol (identical to Taxol-receiving and Taxol-depleted group).

**Fig 2:**
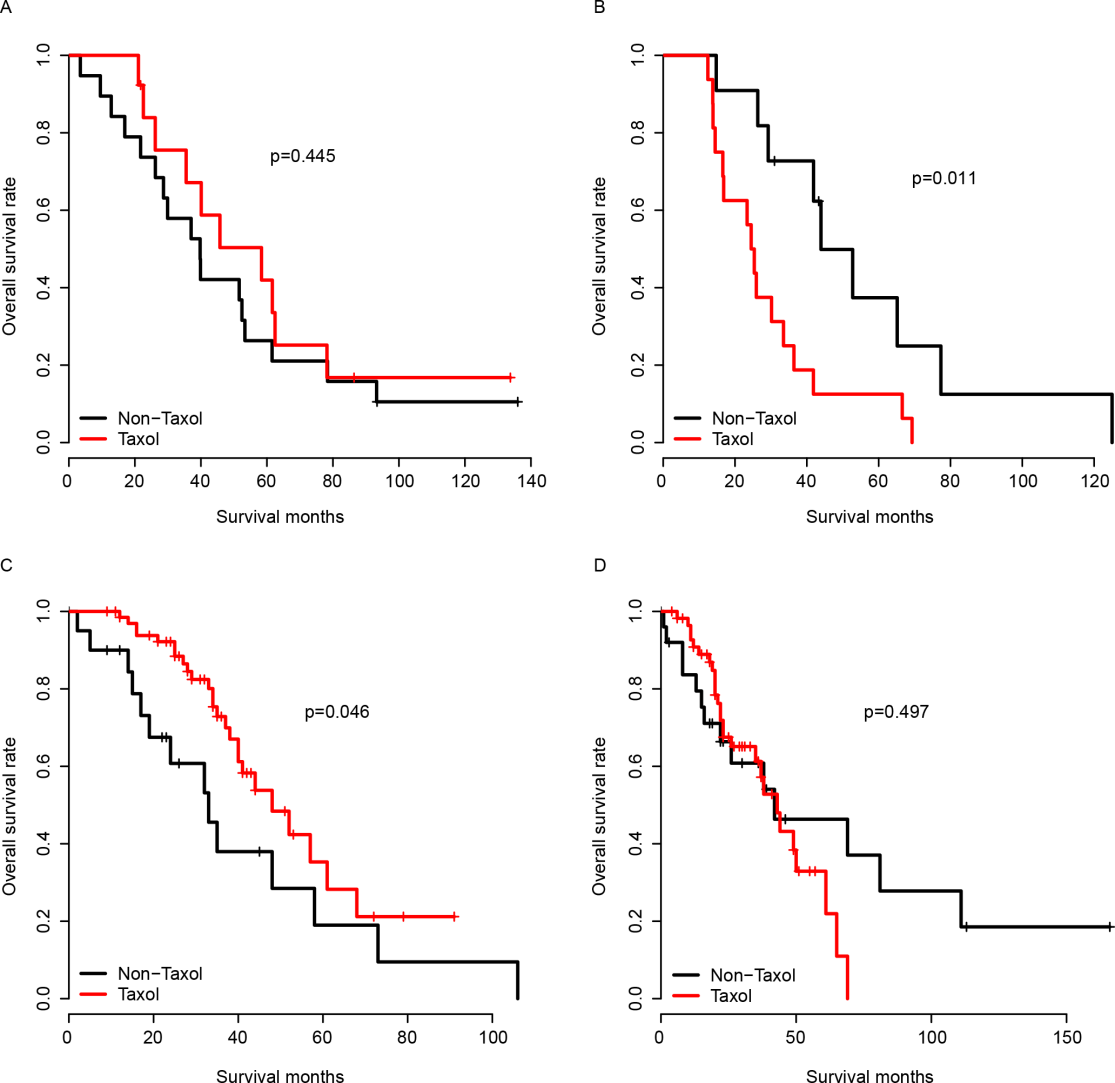
Performance of Taxol score in validation datasets. The performance of Taxol score was validated in the high Taxol score group (A) and low Taxol score group (B) in the GSE63885 cohort and GSE9891 cohort (C-D, high and low Taxol score group).

The performance of the model was first evaluated in the training dataset, which was the TCGA dataset. The survival difference of the high and low Taxol score group was compared using Kaplan-Meier survival analysis. As expected, the patients with a high Taxol score had no significant survival difference between the Taxol-receiving and the Taxol-depleted group (Fig 1A and 1B). The good performance of the model may result from the over-fitness of the training dataset; therefore, validation cohorts were employed to assay the robustness of the model across datasets. GSE63885 and GSE9891 were used for validation due to the explicated Taxol chemotherapy records. The Taxol score of each sample in the validation datasets was calculated using the same coefficients as the training dataset. The Taxol score was divided into a high Taxol score group and a low Taxol score group using the median Taxol score value in each group, as was done for the TCGA cohort. As expected, the patients who underwent Taxol chemotherapy had a better survival than those that did not undergo Taxol treatment in the low Taxol group. However, no significant difference in the survival was observed in the high Taxol group between patients who underwent Taxol chemotherapy or those who did not. This observation was confirmed in both the GSE63885 and GSE9891 groups (Fig 2A and 2D). In summary, the Taxol score is robust in predicting serous ovarian cancer’s response to Taxol in both the training cohort (TCGA) and the validation datasets (GSE63885 and GSE9891).

### Clinical indicators, KEGG pathways and Taxol score

**Fig 3:**
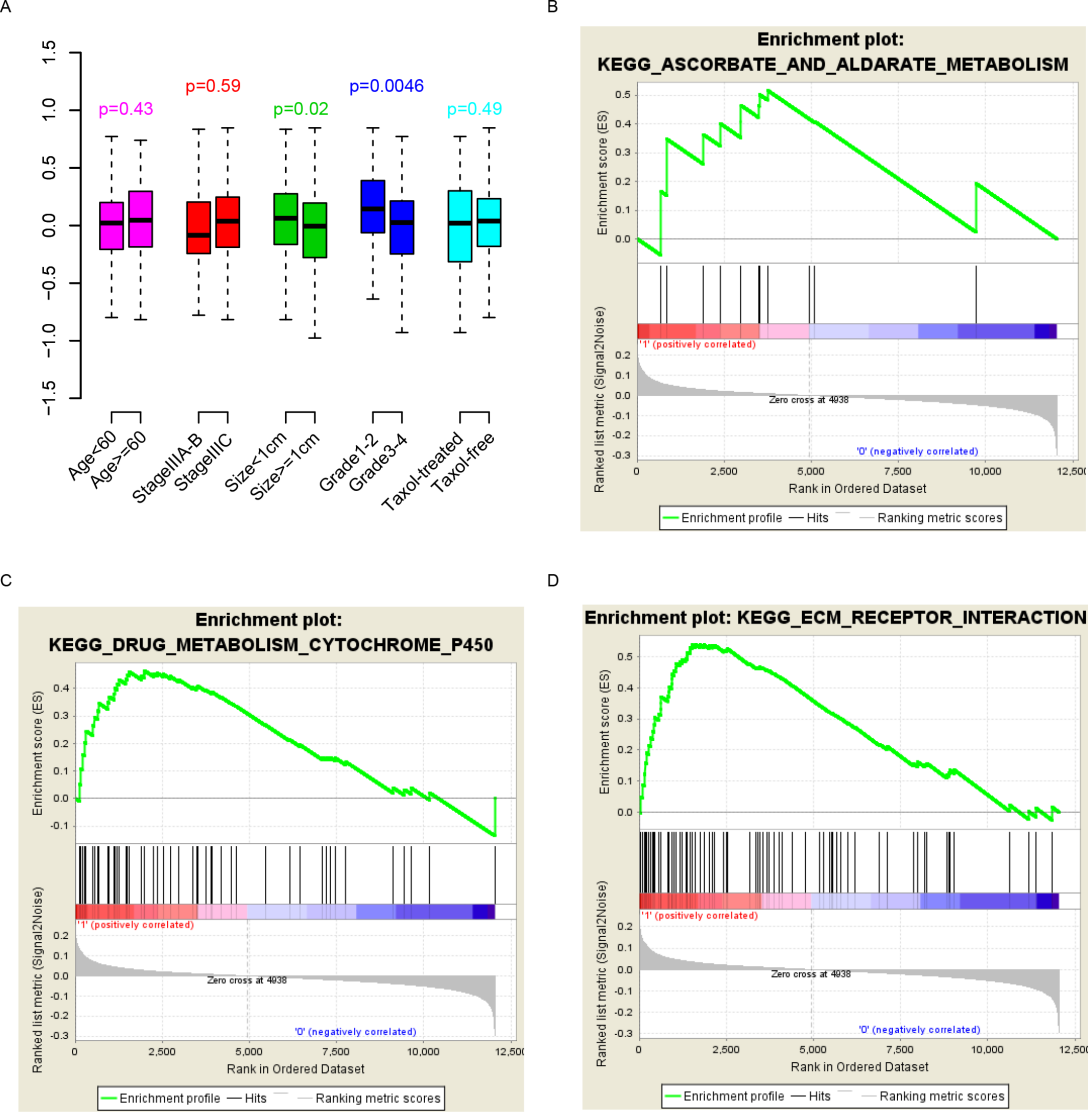
Pathways, clinical indicators and Taxol score. The Taxol score is independent of most indicators (A). Pathways associated with Taxol score were identified.

The correlation between the clinical indicators and the Taxol score was evaluated. As shown in Fig 3A, the Taxol score was independent from age, stage and Taxol treatment, while it was significantly associated with primary tumor size and grade (p = 0.02 and 0.0046, respectively).

To assess the biological status that the Taxol score may reflect, significantly enriched KEGG (Kyoto Encyclopedia of Genes and Genomes) pathways were evaluated using the Gene Set Enrichment Analysis (GSEA) by comparing the expression difference between the high Taxol group and the low Taxol group using the median value. As expected, metabolic signaling pathways including drug metabolism, cytochrome P450, and the ascorbate aldarate metabolism pathway were significantly enriched (Fig 3B and 3E). The ECM receptor signaling pathway was also enriched. In summary, the Taxol score reflects various biological states of serous ovarian cancer and predicts patient response to Taxol.

## Discussion

Taxol is one of the most important chemotherapy drugs for ovarian serous carcinoma. Since the response to Taxol varies across individuals, single molecular biomarkers for drug usage have been developed in the past decades. However, none of these single molecular biomarkers have been used clinically due to their lack of robustness. The heterogeneity of serous ovarian cancer impacts the response to Taxol, which relies on many cellular processes including the cancer subtype and drug metabolism. Therefore, it is reasonable that the response to Taxol cannot be simply predicted using a single gene biomarker. To overcome this weakness, we introduced a Taxol response model and a Taxol score based on gene expression to predict the response of ovarian serous carcinoma to Taxol. The Taxol score successfully predicted the response of Taxol in three independent datasets.

Limitations exist in this work. First, this is a retrospective study. The patients who received Taxol chemotherapy also received other treatment methods, including other chemotherapy drugs; therefore, a bias may have arisen. Additionally, the dosage used was not always clear. Second, the tumor response to Taxol should have been measured by tumor size as it is better than the survival information; however, these data are unavailable.

## Conclusion

The developed Taxol score is robust in predicting the sensitivity to Taxol across serous ovarian samples and cohorts via reflecting various status of serous ovarian carcinoma.

## Supporting information

S1 Table The raw data accessions and URLs. All data is uploaded by third party.
(XLSX)
